# The Exploitation of Carbon Nanomaterials as Electrode Material to Increase the Sensitivity of Germanium Ion Determinations by Stripping Adsorption Voltammetry

**DOI:** 10.3390/ma19010173

**Published:** 2026-01-03

**Authors:** Malgorzata Grabarczyk, Wieslawa Cwikla-Bundyra, Oliwia Siewierska

**Affiliations:** Department of Analytical Chemistry, Institute of Chemical Sciences, Faculty of Chemistry, Maria Curie-Sklodowska University, 20-031 Lublin, Poland; wieslawa.cwikla-bundyra@mail.umcs.pl (W.C.-B.); oliwiasie@wp.pl (O.S.)

**Keywords:** germanium, carbon nanotubes, spherical glassy carbon, electrochemical sensor, adsorptive stripping voltammetry

## Abstract

A highly sensitive and fast procedure for the determination of trace germanium is presented. The carbon nanotubes/spherical glassy carbon electrode (CNTs/SGCE) has been applied for adsorptive stripping voltammetric determination of trace concentrations of Ge(IV) in solution, preceded by complexation with chloranilic acid. Carbon nanomaterials were used for the first time in the voltammetric determination of Ge(IV). The experimental variables such as supporting electrolyte concentration, chloranilic acid concentration, modification of the CNTs/SGCE by forming a bismuth film, and the potential and time for Ge(IV)-chloranilic acid adsorption, as well as instrumental variables on the germanium signal response, were tested. Under optimized conditions, the peak current was found to be proportional to the concentration of Ge(IV) over the range of 0.9 to 30 nmol L^−1^ with R = 0.998. The detection limit, estimated from three times the standard deviation at low Ge(IV) concentration, was about 0.3 nmol L^−1^. Possible interferences were evaluated. Finally, the proposed method was successfully applied for the determination of the total amount of germanium in drinking and river water samples.

## 1. Introduction

The discovery that germanium has semiconductor properties opened up a wide range of applications for it, and this element laid the foundations for the development of modern computers. In the second half of the 20th century, it was largely replaced by much cheaper silicon, but germanium continues to play a key role in various fields of science and technology. Its unique properties make it an invaluable material in the electronics, optics, and photovoltaic industries. Germanium is used in fibre optics, solar cells, and LED technology. In addition, germanium is a compound used in the production of phosphorescent and fluorescent lamps. At the same time, germanium is transparent to infrared radiation and therefore is used in equipment that detects and measures radiation levels. In the military industry, it is used in the production of night vision devices. Germanium dioxide is also used in photographic and microscope lenses. Compared to lead, cadmium, and mercury, germanium is much less toxic to humans, but this does not mean that it has no harmful effects on health. Synthetic germanium compounds in liquid or volatile form can cause irritation to the eyes, throat, lungs, or skin, and in larger quantities can cause liver and kidney disorders, and even death [1,2,3,4,5]. On the other hand, germanium belongs to the group of antioxidants and, together with them, protects the human body against many diseases associated with ageing, including diseases of the musculoskeletal system, inflammation of the spine joints, and rheumatoid arthritis [6]. The literature also suggests that germanium protects against degenerative diseases such as leukaemia, asthma, diabetes, premature ageing, digestive disorders, cardiovascular diseases (coronary heart disease, atherosclerosis, and hypertension), arthritis and spinal inflammation, Parkinson’s disease, epilepsy, as well as cataracts and glaucoma [7]. However, regardless of how we assess this element, it is essential to have information about its occurrence in various environmental samples, preferably at the lowest possible concentrations.

Voltammetry is widely used in environmental monitoring, particularly for the detection of trace metals. This is a technique in which the current flowing through an electrochemical cell is measured as a function of the applied potential. By analysing this current’s response, voltammetry allows the concentration of electroactive analytes in a solution to be determined. In order to determine the lowest possible concentrations, stripping voltammetry is the most commonly used. It involves concentrating the analyte on the working electrode to increase the sensitivity of the measurements [8,9,10]. In the case of Ge(IV) determinations, stripping voltammetry is mainly used in the adsorption stripping voltammetry variant, in which a suitable complexing agent introduced into the solution enables effective concentration of germanium on the working electrode. The voltammetric cell uses a three-electrode system consisting of a working electrode, on which concentration takes place, a reference electrode, and an auxiliary electrode. In the vast majority of procedures for the determination of trace amounts of germanium described in the literature, the working electrodes are based on mercury [11,12,13,14,15,16,17,18,19,20,21]. These include the classic hanging drop mercury electrode [11,12,13,14,15,16,17,18], mercury-coated glassy carbon electrode [19], and less toxic and therefore more environmentally friendly renewable mercury film silver base electrode [20,21]. Due to the toxicity of mercury, research on the voltammetric determination of trace amounts of germanium has focused on the search for a new electrode material that can successfully replace mercury. While various electrode materials have been proposed for the determination of other ions, in the case of germanium, only bismuth can be found in the literature as a substitute for mercury [22,23,24]. In these procedures, a bismuth film was formed on a glassy carbon substrate, generated on both classic glassy carbon electrodes [22,23] and screen-printed carbon-based electrodes [24].

In recent years, a major breakthrough in carbon chemistry has been the introduction of carbon nanomaterials [25,26]. The discovery of carbon nanotubes (CNTs), officially attributed to Sumio Iijima, marked the beginning of a new era in nanotechnology [27]. The unique mechanical, electrical, and thermal properties of nanotubes (they are extremely strong and lightweight and can be better heat conductors than diamond) have revolutionised many fields, including the design of new sensors. The first studies using nanotubes as electrode material were described by Britto [28] and published as early as 1996. Since then, there has been rapid development in the use of carbon nanotubes in electrochemical sensors [29,30,31]. CNTs have unique properties that are extremely important for electrochemical sensors: they have a large specific surface area and high charge transfer capacity, and are hydrophobic and chemically stable. In addition, their presence facilitates electron transfer and increases the signal-to-noise ratio, which results in improved analytical parameters of electrochemical sensors, directly translating into increased sensitivity of determinations [32,33,34]. It is therefore not surprising that there are numerous reports in the literature on voltammetric sensors modified with nanomaterials to increase the sensitivity, selectivity, or rapidity of measurements. These studies are in line with the newest trends, are forward-looking, and are attracting considerable interest [35,36,37]. Therefore, the aim of our research was to use carbon nanotubes to construct an electrochemical sensor that would increase the sensitivity of Ge(IV) determinations using stripping adsorption voltammetry. Carbon nanotubes have already been successfully used to increase the sensitivity of stripping adsorption voltammetry determinations for other elements [38,39,40]. However, in the case of Ge(IV), no such studies had been conducted previously, and they were proposed, carried out, and described for the first time by our research group in this paper. The paper proves that the use of carbon nanotubes as a material for the construction of an electrochemical sensor instead of classic glassy carbon significantly increased the sensitivity of Ge(IV) determinations.

## 2. Materials and Methods

### 2.1. Instrumentation Equipment

The experiments were performed using a μAutolab analyser (Utrecht, The Netherlands). The three-electrode electrochemical cell system was used with the multimode electrode: the working electrode was a multiwall carbon nanotubes/spherical glassy carbon electrode (CNTs/SGCE), the reference electrode was an Ag/AgCl (saturated NaCl), and the auxiliary electrode was a platinum wire. The ultrasonic bath was conducted at Sonic-3, Polsonic, Warsaw, Poland.

### 2.2. Fabrication of the CNTs/SGCE

For the construction of the carbon nanotubes/spherical glassy carbon electrode (CNTs/SGCE), multiwall carbon nanotubes (CNTs) with an outer diameter × inner diameter × length of 10 nm ± 1 nm × 4.5 nm ± 0.5 nm × 3–6 μm and spherical glassy carbon (SGC) powder with particle a size of 0.4–12 µm were used. The CNTs were mixed with epoxy resin in a mass ratio of 1:25 (0.08 g CNTs: 2 g epoxy resin), and the resulting homogeneous mass was heated to 115 °C for 5 min and centrifuged while hot, which allowed the removal of air bubbles that could reduce the performance of the electrode. Next, the resulting mixture of CNTs and epoxy resin was mixed with SGC with a particle size of 0.4–12 µm in a ratio of 2:1. The resulting CNTs-SGC paste was then placed under pressure into a 2 mm diameter hole drilled in the epoxy resin, which formed the electrode housing. For final hardening, the electrode was reheated at 115 °C for 48 h. A copper wire was placed inside the electrode casing to ensure electrical contact. After preparation, the electrode was polished first with coarse-grained sandpaper (P120) and then with fine-grained sandpaper (P2000). Each day before measurements, the CNTs/SGCE was polished on a Buehler polishing block with a 0.3 µm thick aluminium oxide suspension and, after rinsing with distilled water, placed in an ultrasonic bath for approximately 30 s.

### 2.3. Reagents

All reagents used were of analytical-grade purity unless otherwise stated. A stock solution of 1 g L^−1^ Bi(III) and Ge(IV) was obtained from Merck (Darmstadt, Germany). Suprapur CH_3_COOH and NaOH were obtained from Merck. The working solutions of 1 × 10^−4^ mol L^−1^ and 1 × 10^−5^ mol L^−1^ Ge(IV) were prepared by dilution of the stock solution as required. A solution of 1 × 10^−2^ mol L^−1^ chloranilic acid was prepared by dissolving 0.0209 g of the reagent (Fluka- Buchs, Switzerland) in water in a 10 mL volumetric flask. O.D. × I.D. × L multiwall carbon nanotubes were 10 nm ± 1 nm × 4.5 nm ± 0.5 nm × 3~6 μm (Sigma-Aldrich, St. Louis, MO, USA), respectively. Spherical glassy carbon powder with a particle size of 0.4–12 µm was purchased from HTW Hochtemperatur-Werkstoffe GmbH, Thierhaupten, Germany. All solutions were prepared using triply distilled water.

### 2.4. Voltammetric Measurement

Voltammetric measurements were performed in the solution containing 0.1 mol L^−1^ CH_3_COOH as supporting electrolyte, 0.6 mmol L^−1^ chloranilic acid as a complexing agent, and 40 µmol L^−1^ Bi(III), which was used to generate a bismuth film on the CNTs/SGCE. The solution was prepared using triple-distilled water, and its final volume after adding the analysed sample was 10 mL. The voltammetric measurement was performed using the stripping voltammetry method and consisted of the following three stages: generating a bismuth film on the CNTs/SGCE (−1.1 V for 20 s), adsorption of Ge(IV)-chloranilic acid complexes on the bismuth film formed (−0.4 V for 20 s), and recording of the voltammogram as a result of a potential change ranging from −0.2 to −0.8 V. The first two steps were performed in solutions stirred with a magnetic stirrer at a speed of 750 rpm. The third stage was carried out after 10 s of equilibration time in an unmixed solution using the differential pulse technique with a scan rate of 80 mV s^−1^. In order to maintain identical measurement conditions, the electrode was cleaned electrochemically before each measurement using successive potentials of −1.4 V for 10 s and 0.3 V for 10 s. Each measurement was performed three times, and the average value was calculated.

### 2.5. Natural Water Sample Preparation

The developed method was evaluated by determining Ge(IV) in drinking water and river water samples. Drinking water samples were taken from the laboratory, while samples from the Bystrzyca River were collected in clean polypropylene bottles from the Lublin area and pre-treated by filtration through 0.45 μm Millipore membrane filters. Water samples from the Bystrzyca River were collected in November and analysed within a week of collection. During this time, the samples were stored in the form in which they were collected, without acidification. Drinking water was collected immediately prior to analysis. The samples were kept at a temperature of 6 °C. Recovery tests were performed on enriched drinking water and river water samples. Standard solutions were added approximately one hour before analysis to increase the concentration of Ge(IV) in the analysed waters.

## 3. Results

The aim of our work was to use carbon nanotubes to increase the sensitivity of Ge(IV) determinations by stripping adsorption voltammetry using a bismuth film modification. In the procedures for determining Ge(IV) described in the literature to date, the bismuth film was generated on glassy carbon, and chloranilic acid, catechol, or pyrogallol were used as complexing agents [22,23,24]. In our research, we proved that the use of carbon nanotubes as a substrate instead of glassy carbon increases the sensitivity of the determinations. We chose chloranilic acid as the complexing agent because the procedure using it allowed the preparation of a bismuth film in situ in a short time of 20 s [23], while the procedures using catechol and pyrogallol required the preparation of a bismuth film ex situ in a relatively long time of 120 and 300 s, respectively [22,24]. In situ modification methods are preferred over ex situ methods because there is no need to prepare a separate solution to produce the bismuth film. In the in situ method, the electrode is modified with a bismuth film in a solution of the analysed sample enriched with Bi(III) ions. This significantly simplifies the entire measurement procedure and shortens the measurement time. In order to select the most optimal conditions for the determination, we examined the effect of operational parameters such as supporting electrolyte concentration, chloranilic acid concentration, Bi(III) concentration, bismuth film formation potential and time, Ge(IV)-chloranilic acid adsorption potential and time, and instrumental variables.

### 3.1. Effect of Operational Parameters

#### 3.1.1. Influence of Supporting Electrolyte

The presence of Bi(III) in the solution determines the need to use an acidic environment to avoid hydrolysis. In compliance with the literature data, CH_3_COOH was selected as the most optimal supporting electrolyte in our work, allowing for a high germanium signal compared to other acids. In our research, we focused on selecting its concentration. We prepared solutions containing a constant concentration of the other components, i.e., 10 nmol L^−1^ Ge(IV), 0.6 mmol L^−1^ chloranilic acid, and 40 µmol L^−1^ Bi(III), and varying concentrations of CH_3_COOH in the range of 0.02 to 0.2 mol L^−1^. Throughout the entire tested range, the germanium signal reached a similar value. The optimal concentration of the supporting electrolyte was selected as 0.1 mol L^−1^.

#### 3.1.2. Influence of Modification CNTs/SGCE

**Figure 1:**
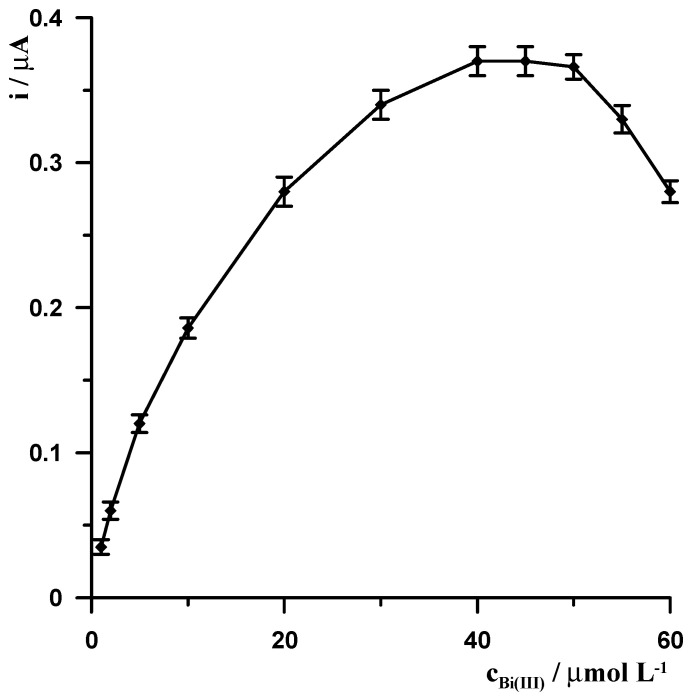
The influence of Bi(III) concentration on the 10 nmol L^−1^ Ge(IV) signal. Constant concentrations of 0.1 mol L^−1^ CH_3_COOH and 0.6 mmol L^−1^ chloranilic acid. Voltammetric measurements were carried out using the following potential change: −1.1 V for 20 s.

The presence of Bi(III) in the solution, similar to chloranilic acid, is necessary to perform the measurement, and in its absence, no germanium peak was observed. This confirms that Ge(IV)-chloranilic acid complexes are not adsorbed directly onto the CNTs/SGCE and that it is necessary to modify the electrode surface by forming a bismuth film. The quality of the bismuth film is primarily influenced by two parameters: the concentration of Bi(III) ions in the solution and the value and duration of the potential at which it is generated. The effect of Bi(III) concentration in the solution on the germanium signal was studied in the range of 1 to 60 µmol L^−1^ using a potential and modification time of −1.1 V and 20 s, respectively. The measurements were performed in a solution containing 10 nmol L^−1^ Ge(IV), 0.6 mmol L^−1^ chloranilic acid, and 0.1 mol L^−1^ CH_3_COOH. As can be seen in Figure 1, with increasing Bi(III) concentration, the germanium signal gradually increases, reaching a constant value in the concentration range of 40–50 µmol L^−1^, and then gradually decreases.

**Figure 2:**
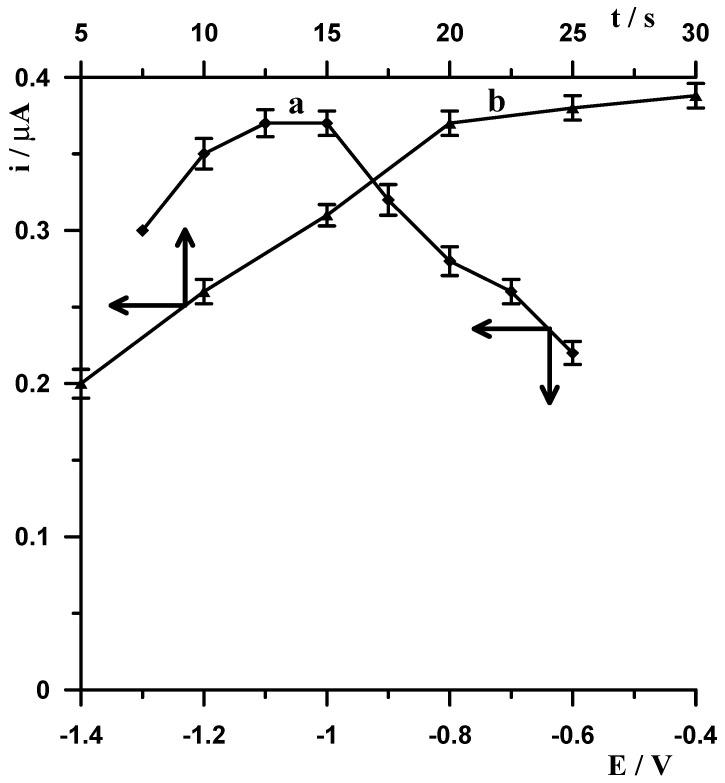
The influence of potential (a) and time (b) of CNTs/SGCE modification on a 10 nmol L^−1^ Ge(IV) signal. Constant concentrations of 0.1 mol L^−1^ CH_3_COOH, 0.6 mmol L^−1^ chloranilic acid, and 40 µmol L^−1^ Bi(III). Voltammetric measurements were carried out using the following potential change: −1.1 V for 20 s.

Next, the potential and modification time were changed at a constant Bi(III) concentration of 40 µmol L^−1^. The potential was changed in the range of −1.3 to −0.6 V and the time in the range of 5 to 30 s. The results obtained are presented in Figure 2. As can be seen, the optimal modification potential is in the range of −1.1 to −1.0 V. At potentials more negative than −1.1 V, the germanium signal decreases, which is probably related to more intense hydrogen evolution. At potentials more positive than −1.0 V, the germanium signal begins to decrease significantly, which clearly indicates that the use of a potential ranging between −1.1 V and −1.0 V allows for the formation of an appropriate bismuth film. This is consistent with the literature data, where it has been proven that −1.0 V is the most suitable potential for generating a bismuth film in germanium determination [22,23]. The mechanism of obtaining a germanium signal under these conditions can be explained as follows: During the electrode modification stage, a bismuth film is formed, and at the same time, Ge(IV) is reduced to its metallic form. When the potential then changes to −0.2 V (at which the voltammogram recording begins), germanium is re-oxidized to Ge(IV), and because chloranilic acid is present in the solution, a Ge(IV)-chloranilic acid complex is immediately formed in the electrode layer, which is reduced to its metallic form during the recording of the voltammogram. This process is indicated by the fact that without the presence of chloranilic acid, no peak is observed on the voltammogram, as described in Section 3.1.4.

With increasing modification time, the germanium signal increases to 20 s, and then its increase becomes insignificant (Figure 2(b)). The optimal modification conditions were selected as a concentration of 40 µmol L^−1^ Bi(III) and a potential and time of −1.1 V and 20 s, respectively.

#### 3.1.3. Influence of Adsorption of Ge(IV)-Chloranilic Acid

**Figure 3:**
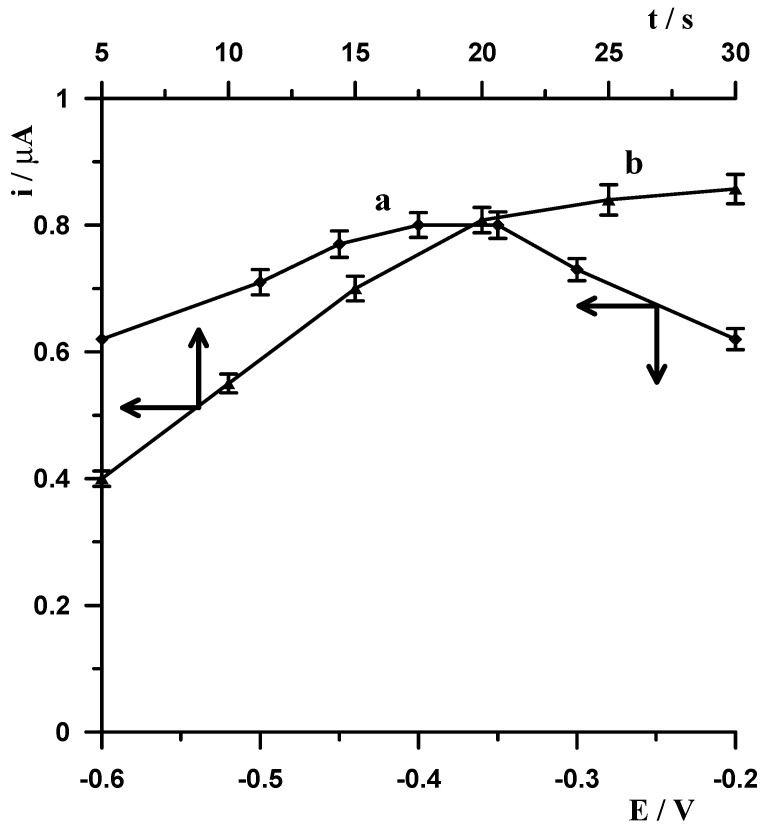
The influence of potential (a) and time (b) of Ge(IV)-chloranilic acid adsorption on the 10 nmol L^−1^ Ge(IV) signal. Constant concentrations of 0.1 mol L^−1^ CH_3_COOH, 0.6 mmol L^−1^ chloranilic acid, and 40 µmol L^−1^ Bi(III). Voltammetric measurements were carried out using the following potential changes: −1.1 V for 20 s, −0.4 V for 20 s.

Based on the literature data and after performing additional experiments, it was found that introducing an additional stage after the electrode modification stage, during which the adsorption of Ge(IV)-chloranilic acid complexes occurs, increases the sensitivity of the germanium signal. Therefore, the influence of the potential at which adsorption occurs on the bismuth film of Ge(IV)-chloraniclic acid complexes was also evaluated (Figure 3(a)). The effect of the adsorption potential on the stripping peak current of the germanium peak was studied over the −0.6 to −0.2 V range. The peak current was maximum between −0.4 and −0.35 V and gradually decreased below and above these values. At potentials more negative than −0.4 V, the germanium signal decreased, which is related to the fact that at potentials below −0.4 V, Ge(IV) reduction begins to occur, thereby decreasing the efficiency of germanium adsorption in the form of the Ge(IV)-chloranili acid complex.

Then, the adsorption time was examined in the 5–30 s range (Figure 3(b)). Peak current increases when the accumulation time is increased from 5 to 20 s, which indicates that Ge(IV)-chloranilic acid complexes are rapidly adsorbed, and then, at longer adsorption times, the peak current increased only slightly while the peak became broad, losing selectivity. The optimum adsorption conditions for the Ge(IV)-chloranilic acid complex were selected as a potential of −0.4 V and a time of 20 s.

#### 3.1.4. Influence of Chloranilic Acid

**Figure 4:**
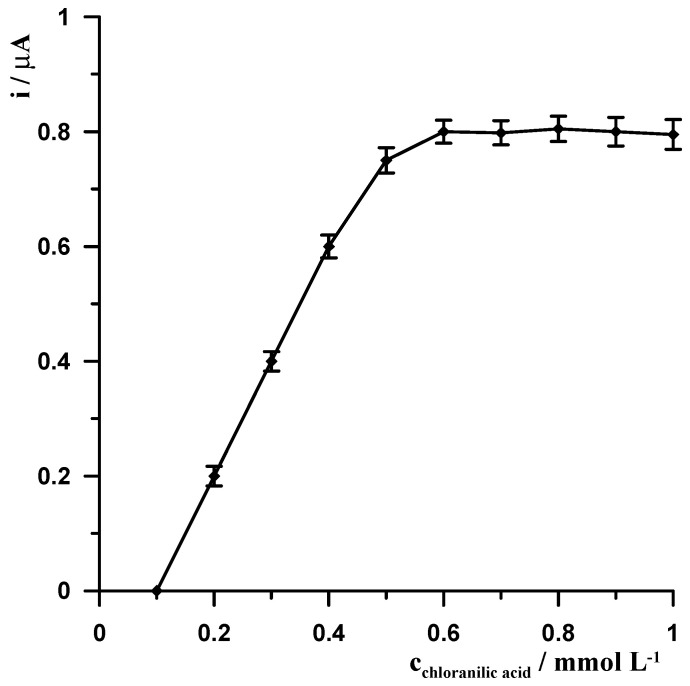
The influence of chloranilic acid concentration on the 10 nmol L^−1^ Ge(IV) signal. Constant concentrations of 0.1 mol L^−1^ CH_3_COOH and 40 µmol L^−1^ Bi(III). Voltammetric measurements were carried out using the following potential changes: −1.1 V for 20 s, −0.4 V for 20 s.

In order for Ge(IV) to accumulate on the working electrode, it must be in the form of electrochemically active complexes that undergo efficient adsorption on the electrode. Various complexing agents dedicated to this purpose have been described in the literature, such as gallic acid, 3,4-dihydroxybenzaldehyde, pyrogallol, pyrocatechol violet, catechol, and chloranilic acid [11,12,13,14,15,16,17,18,19,20,21,22,23,24]. As proven in [23], very effective adsorption occurs on the bismuth surface when Ge(IV) is complexed with chloranilic acid, which is why it was chosen for our procedure. The results of the study are presented in Figure 4. In the absence of the ligand, no germanium peak was observed; a slight signal began to appear in the presence of at least 0.2 mmol L^−1^ chloranilic acid. The germanium signal then increased to a concentration of 0.6 mmol L^−1^ and remained constant up to 1 mmol L^−1^ chloranilic acid. The experiments were carried out using a solution with constant concentrations of the other components: 10 nmol L^−1^ Ge(IV), 40 µmol L^−1^ Bi(III) and 0.1 mol L^−1^ CH_3_COOH. The optimal ligand concentration was selected as 0.6 mmol L^−1^.

### 3.2. Analytical Characterization

**Figure 5:**
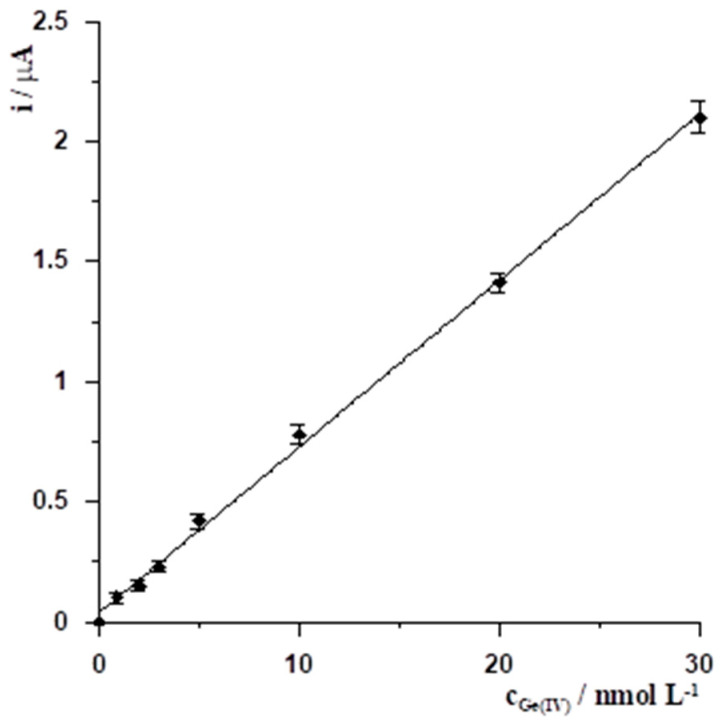
The Ge(IV) calibration curve obtained for its concentration ranging from 0.9 to 30 nmol L^−1^ with an accumulation time of 20 s for Ge(IV)-chloranilic acid complexes, yielding R = 0.998.

**Figure 6:**
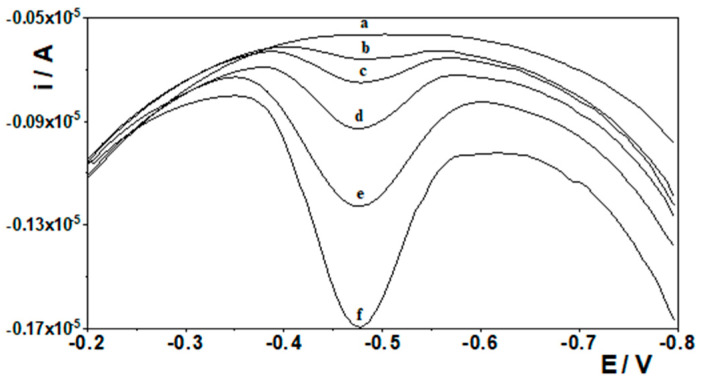
Voltamperograms for the calibration curve recorded for low Ge(IV) concentrations: (a) blank, (b) 0.9 nmol L^−1^, (c) 2 nmol L^−1^, (d) 3 nmol L^−1^, (e) 5 nmol L^−1^, and (f) 10 nmol L^−1^.

A series of voltammograms for increasing Ge(IV) concentration with corresponding calibration curves were recorded. Measurements were carried out using a solution with a constant composition of 0.1 mol L^−1^ CH_3_COOH, 0.6 mmol L^−1^ chloranilic acid, and 40 µmol L^−1^ Bi(III). Voltammetric measurements were carried out using the stripping voltammetry method with the following potential changes: −1.1 V for 20 s, −0.4 V for 20 s. Finally, the voltammetric curve as a result of a potential change ranging from −0.2 to −0.8 V was recorded. The calibration plot obtained was linear from 0.9 to 30 nmol L^−1^ and obeyed the equation y = 0.07x + 0.03, where y and x are the peak current (µA) and the Ge(IV) concentration (nmol L^−1^), respectively. The linear correlation coefficient was R = 0.998. The calibration curve and the voltammograms obtained for low concentrations of Ge(IV) are presented in Figure 5 and Figure 6, respectively.

For each concentration, the voltammogram was recorded three times. The total measurement time, including electrochemical cleaning of the electrode prior to the final measurement, was approximately 60 s. The detection limit (LOD) of the proposed procedure was 0.3 nmol L^−1^ for 20 s Ge(IV)-chloranilic acid adsorption. LOD was calculated from the equation 3 s/m (s is the standard deviation of the peak current for a low Ge(IV) concentration and m is the slope of the calibration curve). The quantification limit (LOQ) of Ge(IV) determination for 20 s of adsorption was calculated to be 1 nmol L^−1^. LOQ was estimated based on the equation 10 s/m. Table 1 presents a comparison of the analytical parameters of the proposed procedure with those obtained using other bismuth-modified electrodes described in the literature for the determination of Ge(IV) [22,23,24]. As you can see, the detection limit obtained in this work is several times lower compared to when glassy carbon was used as the substrate for modification [23], which confirms that the use of carbon nanotubes instead of glassy carbon as a substrate for the formation of bismuth film allows for the detection limit of the determined depolariser to be lowered.

### 3.3. Tolerance to Interfering Species

**Figure 7:**
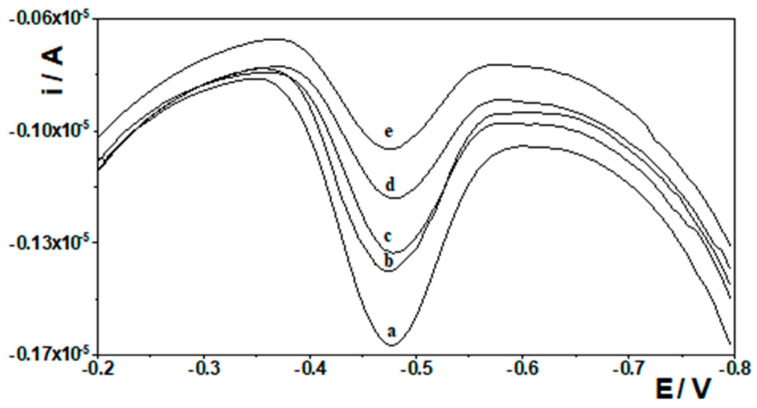
Example voltamperograms of 10 nmol L^−1^ Ge(IV) recorded in the absence of interfering species (a) and in the presence of (b) 100 nmol L^−1^ Mo(VI), (c) Se(IV), (d) Hg(II), and (e) Ti(IV).

Possible interferences in the determination of Ge(IV) using the bismuth film-modified CNTs/SGCE were examined under optimum experimental conditions. The number of metal ions that could potentially interfere were examined: Al(III), Ca(II), Co(II), Cd(II), Cr(III), Cr(VI), Cu(II), Fe(III), Ga(III), Hg(II), In(III), Mg(II), Mn(II), Mo(VI), Ni(II), Pb(II), Sb(III), Se(IV), V(V), Ti(IV), and Zn(II). The studies were performed using a solution with a constant Ge(IV) concentration of 10 nmol L^−1^, to which increasing concentrations of interfering ions were added. For each interfering ion, the studies were performed separately. The concentrations of the interfering ions were 0.02, 0.05, 0.1, 0.2, 0.5, 1, and 2 µmol L^−1^, respectively. For each of these concentrations, the germanium signal was recorded in order to compare it with the germanium signal obtained without the presence of the interfering species. The criterion adopted was that a foreign ion does not interfere with the determination of Ge(IV) if its presence does not cause changes greater than 5% in the analytical signal of germanium. The results obtained are presented in Table 2. As can be seen, the most interfering ions among those tested were Hg(II), Mo(VI), Se(IV), and Ti(IV), which confirms the conclusions presented in earlier studies in which determinations were carried out using a bismuth film-modified glassy carbon electrode. Representative voltammograms recorded in the presence of higher concentrations of the most interfering species are presented in Figure 7.

Another factor that commonly pollutes natural waters is ethylenediaminetetraacetic acid (EDTA). This compound is non-biodegradable and prone to accumulation in environmental waters, where it enters as a result of human activity. It is commonly used, among other things, as an ingredient of detergents, in the production of paper and textiles, and in pharmaceuticals. Based on data from the World Health Organisation (WHO), it is assumed that maximum EDTA concentrations of 0.2 µmol L^−1^ normally occur in sewage [21]. The tests carried out in our work showed that the presence of EDTA in such and even higher concentrations does not interfere with the measurements, which makes the procedure we propose suitable for environmental water analysis.

### 3.4. Analytical Application of the CNTs/SGCE

**Figure 8:**
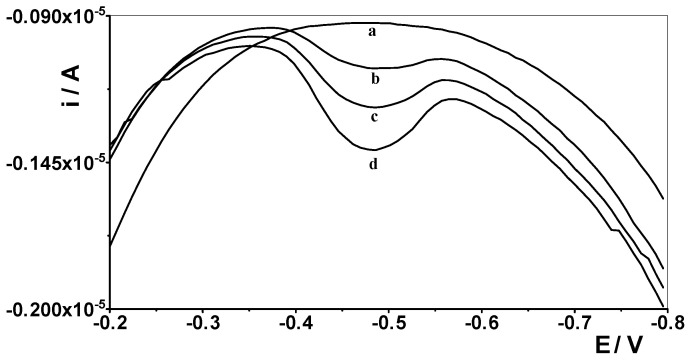
Differential pulse voltammograms obtained in the course of germanium AdSV determination in the Bystrzyca River water. Bystrzyca River water diluted five times (a). Bystrzyca River water spiked with 10 nmol L^−1^ Ge(IV): (b) diluted five times; (c) as (b) + 2 nmol L^−1^ Ge(IV); (d) as (b) + 2 nmol L^−1^ Ge(IV).

The proposed procedure was used to determine the recovery of Ge(IV) from drinking water and river water after introducing various concentrations of germanium into them. Three measurements were performed for each sample, and recoveries were calculated using the standard addition method. The results obtained are presented in Table 3, and the voltammograms obtained in the course of analysis of the Bystrzyca River water are presented in Figure 8. The recovery results obtained were satisfactory for both the spiked samples, which confirms the analytical application of the CNTs/SGCE.

## 4. Conclusions

In our work, we proposed for the first time the use of carbon nanotubes as electrode material, which, thanks to their large surface area and small spatial dimensions, enabled an increase in the sensitivity of trace concentration determinations of Ge(IV) ions. Carbon nanotubes were mixed with spherical glassy carbon, and the electrode obtained on the basis of this mixture—CNTs/SGCE—was modified with a bismuth film, which ensured effective accumulation of Ge(IV)-chloranilic acid complexes. This resulted in a low detection limit of 0.3 nmol L^−1^ for Ge(IV) with a short total measurement time of 60 s. This confirmed that carbon nanomaterials are a desirable electrode material in voltammetry and can achieve low detection limits. The advantages of the CNTs/SGCE sensor include easy cleaning, thanks to the use of an appropriate sequence of potentials before each measurement. Another advantage of the proposed sensor is that it ensures a stable Ge(IV) signal even in the presence of an excess of foreign ions and EDTA in the solution. The majority of the foreign ions tested did not affect the germanium signal, even when present in 100- or 200-fold excesses. The most interfering ions were Mo(VI), Se(IV), Hg(II), and Ti(IV), which caused a decrease in the germanium signal, even at a 10-fold excess. The successful practical application of the proposed sensor for Ge(IV) determination in natural water samples, such as river water and drinking water, seems promising in environmental measurements.

## Figures and Tables

**Table 1:** Comparison of analytical parameters obtained using bismuth film-modified electrodes for Ge(IV) determination. The works are listed in order of decreasing LOD.

Type of Electrode Modified with Bismuth Film	Modification Time [s]	Method of Modification	Accumulation Time of Ge(IV) Complex [s]	Complexing Agent	Linear Range [nmol L^−1^]	LOD [nmol L^−1^]	Ref.
glassy carbon electrode	20	in situ	30	chloranilic acid	3–150	1.2	[23]
glassy carbon electrode	300	ex situ	540	pyrogallol	6.9–230	0.83	[22]
screen-printed electrode	no data	ex situ	30	catechol	5–90	0.8	[24]
CNTs/SGCE	20	in situ	20	chloranilic acid	0.9–30	0.3	this work

**Table 2:** Tolerance levels of interfering species in the determination of 10 nmol L^−1^ Ge(IV) performed under the recommended conditions.

Foreign Ions	Tolerance Level (mol L^−1^)
Al(III), Ca(II), Cr(III), Cr(VI), Cu(II), Ga(III), In(III), Mg(II), Mn(II), V(V), Zn(II)	2 × 10^−6^
Co(II), Cd(II), Fe(III), Ni(II), Pb(II), Sb(III)	1 × 10^−6^
Mo(VI), Se(IV)	2 × 10^−7^
Hg(II), Ti(IV)	5 × 10^−8^

**Table 3:** The results of Ge(IV) determination in water samples spiked with different concentrations of Ge(IV) (n = 3).

Water Sample	Concentration of Ge(IV) [nmol L^−1^]		Recovery [%]
	Spiked	Found	
Drinking water	10	9.87 ± 0.47	98.7
	20	19.36 ± 1.05	96.8
Bystrzyca River water	10	9.71 ± 0.58	97.1
	20	18.68 ± 1.25	93.4

## Data Availability

The original contributions presented in the study are included in the article; further inquiries can be directed to the corresponding author.
